# Effects of Simulated Microgravity and Hypergravity Conditions on Arm Movements in Normogravity

**DOI:** 10.3389/fncir.2021.750176

**Published:** 2021-12-14

**Authors:** Marko Jamšek, Tjaša Kunavar, Gunnar Blohm, Daichi Nozaki, Charalambos Papaxanthis, Olivier White, Jan Babič

**Affiliations:** ^1^Laboratory for Neuromechanics and Biorobotics, Jožef Stefan Institute, Department of Automatics, Biocybernetics and Robotics, Ljubljana, Slovenia; ^2^Jožef Stefan International Postgraduate School, Ljubljana, Slovenia; ^3^Centre for Neuroscience Studies, Queen’s University, Kingston, ON, Canada; ^4^Division of Physical and Health Education, Graduate School of Education, The University of Tokyo, Tokyo, Japan; ^5^INSERM UMR1093-CAPS, Université Bourgogne Franche-Comté, UFR des Sciences du Sport, Dijon, France; ^6^Faculty of Electrical Engineering, University of Ljubljana, Ljubljana, Slovenia

**Keywords:** microgravity, hypergravity, arm kinematics, robot assisted training, parabolic flight, exoskeletons

## Abstract

The human sensorimotor control has evolved in the Earth’s environment where all movement is influenced by the gravitational force. Changes in this environmental force can severely impact the performance of arm movements which can be detrimental in completing certain tasks such as piloting or controlling complex vehicles. For this reason, subjects that are required to perform such tasks undergo extensive training procedures in order to minimize the chances of failure. We investigated whether local gravity simulation of altered gravitational conditions on the arm would lead to changes in kinematic parameters comparable to the full-body experience of microgravity and hypergravity onboard a parabolic flight. To see if this would be a feasible approach for on-ground training of arm reaching movements in altered gravity conditions we developed a robotic device that was able to apply forces at the wrist in order to simulate micro- or hypergravity conditions for the arm while subjects performed pointing movements on a touch screen. We analyzed and compared the results of several kinematic parameters along with muscle activity using this system with data of the same subjects being fully exposed to microgravity and hypergravity conditions on a parabolic flight. Both in our simulation and in-flight, we observed a significant increase in movement durations in microgravity conditions and increased velocities in hypergravity for upward movements. Additionally, we noted a reduced accuracy of pointing both in-flight and in our simulation. These promising results suggest, that locally simulated altered gravity can elicit similar changes in some movement characteristics for arm reaching movements. This could potentially be exploited as a means of developing devices such as exoskeletons to aid in training individuals prior to undertaking tasks in changed gravitational conditions.

## Introduction

Eye-hand coordination is necessary for many everyday tasks that involve grabbing or manipulating objects around us. High proficiency in eye-hand coordination is especially crucial for humans controlling vehicles or complex systems or performing piloting tasks (Paloski et al., 2008). However, human sensorimotor control has evolved in the Earth’s environment where all movements are influenced by the gravitational force (Fisk et al., 1993; Smetanin and Popov, 1997). Changes or the mere absence of this environmental force can drastically affect the performance of arm reaching movements especially in early exposure to the novel environmental dynamics as was observed in force field experiments (Lackner and Dizio, 1994; Shadmehr and Moussavi, 2000) or microgravity (Papaxanthis et al., 2005). Adaptation and training for exposure to changing environmental conditions are critical especially in space flight exploration where astronauts must be prepared on how to operate and complete a multitude of tasks in periods of hypergravity as well as microgravity.

Designing efficient training procedures or simulation environments requires good knowledge of how microgravity and hypergravity affect human sensorimotor control. Several studies already explored the effect of these environmental conditions on arm reaching kinematics in space, during parabolic flights, and in human centrifuges as well as the effects after returning to earth from space (Kornilova et al., 2016). However, the results are not always consistent from study to study. In some cases, the authors found a reduction of movement duration in microgravity compared to normogravity (Mechtcheriakov et al., 2002; White et al., 2008; Crevecoeur et al., 2010) while in other cases, no significant changes were detected (Bringoux et al., 2012; Macaluso et al., 2017). In general, it has been shown that the new environmental conditions negatively affect the accuracy or pointing position of subjects as compared to normogravity (Bock et al., 1992; Fisk et al., 1993). However, in studies where the subjects were trained cosmonauts, no changes in accuracy were found when comparing microgravity and normogravity conditions (Berger et al., 1997; Mechtcheriakov et al., 2002). These discrepancies in results could be just due to the limited number of subjects included in these studies or they might indicate that trained subjects are indeed able to perform better in such environments.

One option for training astronauts before space missions is the use of parabolic flights. However, these are very expensive, the exposure time to microgravity is very limited, and the gravitational environment varies. The other more established method to prepare astronauts for performing tasks in microgravity, is underwater training in neutral buoyancy (Bolender et al., 2006). Some studies explored the effects of neutral buoyancy on sensorimotor control and also compared the effect to those observed in microgravity (Macaluso et al., 2017). However, training underwater does not affect the vestibular signals in the same way as microgravity. Furthermore, faster movements generate additional viscous resistance forces that are not present in microgravity. An alternative for simulating microgravity would be the use of weight support systems to locally remove the effect of gravity on a limb of the individual. There has been some work on exploring kinematic features with weight support systems, but mainly for rehabilitation purposes for stroke patients (Prange et al., 2009; Coscia et al., 2014). In these studies, no significant differences were found for movement duration and movement symmetry. However, these studies did not investigate movements with or against gravity and therefore cannot be compared with other studies investigating movement kinematics in space or parabolic flights.

In this study, we investigated whether simulation of hyper- and microgravity conditions locally on the arm could be a feasible approach for on-ground training of arm reaching movements in altered gravity conditions. To achieve this, we developed a low friction robotic device that was able to apply forces at the wrist in order to simulate micro- or hypergravity conditions for the arm while subjects performed pointing movements on a touch screen. We compared the results of various kinematic parameters using this system with data from a parabolic flight where the same subjects were fully exposed to micro- and hypergravity conditions.

## Materials and Methods

The study was performed during the 142nd CNES parabolic flight campaign that included three flights over 3 days. The flights were composed of 31 parabolic maneuvers. Each maneuver consisted of three phases: 20 s of hypergravity (1.8 *g*, pull-up phase) followed by 22 s of microgravity (0 *g*) before a second period of 20 s of hypergravity (1.8 *g*, pull-out phase). A more technical and in-depth description of the parabolic flight maneuvers is presented by Pletser et al. (2016). The second part of the study was performed on the ground, where we simulated micro- and hypergravity conditions for the arm of the subjects with a robotic system.

### Subjects

Nine right-handed subjects (seven males, two females) participated in the study (age: 29.8 ± 7.4 years, height: 176.0 ± 10.8 cm, weight: 71.0 ± 15.7 kg). No subject reported any musculoskeletal disorders. To avoid motion sickness during the parabolic flight, all subjects received a personalized dose of scopolamine prior to the flight. It has been previously shown, that use of scopolamine does not interfere with sensorimotor control (Ritzmann et al., 2016). None of the subjects had prior microgravity or hypergravity experience. They were all naive with respect to the specific purpose of this experiment. All subjects gave their informed consent to participate in the study, stored by the Caen University Hospital. The experiment was conducted in accordance with the Declaration of Helsinki, procedures were approved by the French National Ethics committee (2018-A03379-46) and authorized by the ANSM (French National Agency for Biomedical Security).

### Experimental Setup

**Figure 1:**
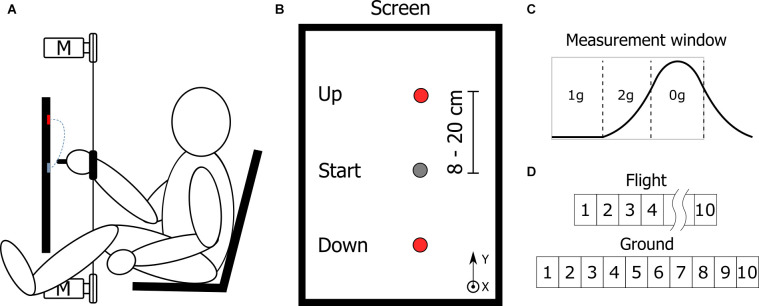
**(A)** Profile view of the experimental setup and **(B)** view of the screen indicating start (Gray) and end target (Red) locations. The orientation of the coordinate system is marked indicating the positive y coordinate pointing up and the x coordinate pointing out from the screen towards the subject. **(C)** The measurement window during each parabola consisted of 20 s of normogravity followed by 20 s of hypergravity and finally 22 s of microgravity. **(D)** The number of parabolas per subject. The parabolas from 5 to 9 during flight were part of another study.

The subjects were seated in a chair (Figure 1A) positioned in front of a touch screen display (ProLite T2435MSC-B2, Iiyama, Hoofddorp, The Netherlands) oriented in portrait mode (display size 521 mm × 293 mm) as depicted in Figure 1B. The middle of the screen was positioned at a height of 750 mm so that subjects could comfortably reach the top and bottom of the screen. To prevent trunk displacement during the task, subjects were securely strapped to the chair using a four-point harness. The subject’s legs were positioned on the outsides of the screen with the ankles strapped firmly in place. This was done in order to prevent involuntary leg movements during microgravity phases.

During the experiment, the subject’s right wrist was strapped with a Velcro strap to a non-stretchable string (Dyneema^®^ 1.5 mm, YSM and Partners, Dobra, Poland) which was connected to two motors (EMMS-AS-55-S-TM, Festo, Esslingen, Germany), positioned above and below the center of the screen. The distance between the motors was 1.5 m. To prevent string slack, a constant pretension force of 10 N was applied by both motors in the opposite direction. This allowed for unobstructed vertical movements of the arm while maintaining a constant connection of the wrist to the motors. In the trials performed on the ground, the motors were used to apply a force at the subject’s wrist, mimicking the gravitational effects of microgravity or hypergravity conditions at the shoulder joint. To simulate microgravity, the motors applied a constant force in the upward direction, whereas to simulate hypergravity conditions, the force was applied in the downward direction. In simulated normogravity conditions, no additional force was applied to the wrist, only the pretension was used in order to prevent string slack. The force used was subject-specific and was measured beforehand (18.6 ± 4.8 N). The force controller to control the motors was running on a real-time computer at a rate of 1 kHz and in a closed loop. To monitor the kinematics of the subjects, a motion capture system consisting of three cameras was used (Vicon, Yarnton, United Kingdom). A marker was placed on the stylus used for performing the task. The acquisition frequency for the kinematics was 100 Hz.

To collect data of muscle activity, EMG electrodes (SX230 sensor, Biometrics Ltd, Newport, UK) were placed on the skin following SENIAM recommendations (Hermens et al., 2000) on the Anterior and Posterior Deltoid, Trapezius, and Pectoralis. Raw EMG signals were recorded at a frequency of 1,000 Hz on a Sensoray Model 526 (Sensoray, Tigrad, USA).

### Task

Subjects had to successfully point to a target presented on the screen in front of them. To perform the task, subjects used a tactile stylus held by the thumb, index, and middle finger. The starting position was indicated by a gray circle of a diameter of 60 mm and was located approximately at the shoulder height. After a random delay of 0–500 ms, the end target (diameter of 20 mm) was presented to the subject. The seven possible end target positions were 8, 10, 12, 14, 16, 18, and 20 cm from the start target either vertically up or down. To prevent anticipation, the sequence of displayed targets was randomized. The subjects were instructed to perform the task as fast and as accurately as possible. On average subjects performed 20.8  ±  4.3 trials per each parabola.

### Protocol

The whole experiment consisted of one in-flight session and one simulation session performed on the ground. In the in-flight session, each subject performed the experiment for 10 parabolas. This included 20 s before the parabola (normogravity), 20 s during hypergravity, and 22 s during microgravity (Figure 1C). During other parts of the flight, the subjects were instructed to rest their arm on their right leg.

In the simulation session (carried out one day after all the flight sessions), each subject performed the same task for the duration of 10 simulated parabolas. Each simulated parabola consisted of 20 s with the motors inactive (normogravity), followed by 20 s with the motors active and applying a downward force to the wrist simulating hypergravity and finally, 22 s with the motors active and applying an upward force to the wrist, simulating microgravity. After each simulated parabola, the subjects rested their arm. In the simulation session, only seven out of nine subjects participated in the experiment.

As indicated in Figure 1D, only five parabolas during flight were used for the analysis in this study. The parabolas five-nine were part of another study where the subjects received an assistive force at the wrist to compensate for the gravitational changes experienced during the parabolic flight and thus simulated constant normogravity conditions for the arm performing the experiment.

For a concise representation of different conditions throughout this article, we will refer to the in-flight phases of normogravity, microgravity, and hypergravity with labels (1g, 0g, and 2g) respectively. For the trials performed on the ground, we will refer to them as simulated normogravity, microgravity, and hypergravity conditions with labels (1g S, 0g S, and 2g S). We use the term simulated normogravity, even though during these trials we did not simulate any additional forces. The colors used in the figures of results are coherent between actual or simulated conditions, where green denotes 1g/1g S conditions, blue denotes 0g/0 gS conditions and red denotes 2g/2g S conditions.

### Data Processing and Analysis

Data from the touch screen were used to calculate movement duration and accuracy. The trial started when the stylus moved away from the screen and ended when it touched the screen again. Movement duration was defined as the time from the start to the end of one trial. To assess accuracy, the pointing position relative to the target was calculated as the vertical distance between the center of the target and the position where the stylus touched the screen. We refer to these results as deviations from the targets, where a positive deviation represents a hit above the target location whereas a negative deviation represents a hit under the target location in the coordinate frame defined in Figure 1B.

Marker positions were interpolated for missing data and low pass filtered with 2nd order zero-phase lag Butterworth filter (10 Hz cut-off frequency). Trials with excessive missing data or data with clear outliers were manually removed from the kinematic analysis. Position data were used to calculate displacement in the x direction, the velocity profiles, maximum velocity, and relative time-to-peak-velocity (TPV) as a measure of movement asymmetry. Motion trajectories were plotted with normalized positions in the vertical direction and averaged positions in the horizontal direction.

EMG signals were band-pass filtered (zero phase lag, 2nd order Butterworth filter with cut-off frequencies of 20 and 350 Hz) and full-wave rectified. The envelope of the signal was extracted with a moving average window of 100 samples. Finally, the signals were normalized to the mean of the 1 g condition and integrated over time for each trial to express the magnitude of muscle activity (normalized iEMG).

All data processing and generation of data figures were performed in Matlab (Mathworks, Natick, MA, USA).

#### Statistical Analysis

To compare parameters across different conditions, we conducted a linear mixed models analysis with three gravitational conditions (1g, 0g, 2g) × two simulation conditions (real, simulated) × seven targets statistical design where the subjects were included as random effects. The analysis was conducted in R (R Core Team, 2020) with the nlme (Pinheiro et al., 2020) and multcomp (Hothorn et al., 2008) packages. We checked that the residuals of the fitted model were normally distributed. We performed all analyses separately for the upwards and downwards movements. We report only the main effects of gravity and simulation as well as the interaction effect of gravity × simulation. *Post hoc* tests with Bonferroni correction were conducted to determine significant differences between specific conditions. To specifically determine the effects of microgravity on task variables, we compared (1g–0g and 1g S–0g S) and also directly compared the parameters with simulated microgravity with actual microgravity on the plane (0g S–0g). The same comparisons were conducted for changes of movement parameters in hypergravity (1g–2g, 1g S–2g S, 2g S–2g). The final comparison included the two conditions of normogravity (1g S–1g). The level of statistical significance was set at 0.05. We adopted conventional statistical significance labels: * <0.05, ** <0.01, *** <0.001.

## Results

First, we present the results of the pointing accuracy which is the main task outcome. We then present movement kinematics. Starting with movement duration, velocity profiles, and maximum velocity, followed by the shape of the trajectories and relative time to peak velocity. Finally, we present the results of muscle activation during the different conditions.

The results for each parameter are structured in the same form where the results for downward movements are presented first followed by results for upward movements. For each direction, we first report the effects of gravity, simulation, and the interaction of gravity × simulation. Then, individual differences and results of *post hoc* tests are first presented for microgravity conditions (0*g*), followed by hypergravity (2*g*), and finally, we report if there were any differences between normogravity conditions (1*g*) during flight vs. the simulation trials. We do not report the effects of target positions, however as mentioned in subsection “Statistical Analysis”, the statistical analysis was performed taking into account the different target locations.

### Task Outcome: Accuracy

**Figure 2:**
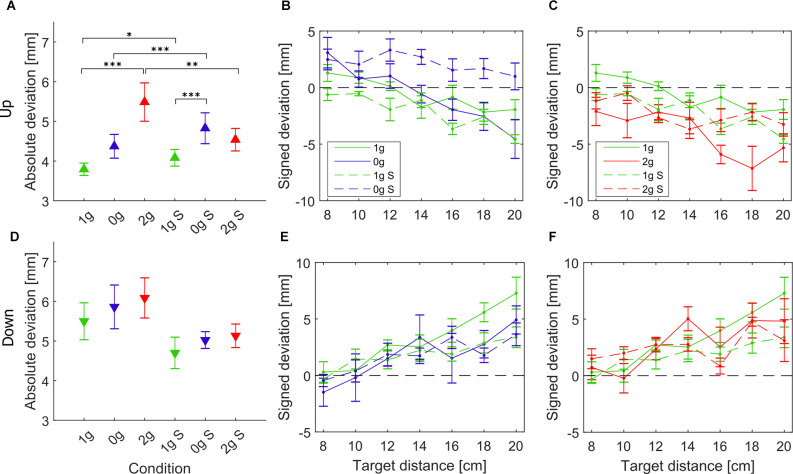
Absolute deviations of the pointing position averaged for all subjects and grouped per condition for upward movements **(A)** and downward movements **(D)**. Signed deviations for every target in microgravity **(B,E)** and hypergravity **(C,F)** for upward and downward movements, respectively. Downward-pointing triangles denote mean values for downward movements, upward-pointing triangles denote mean values for upward movements, the whiskers denote the standard error of the mean. Green represents normogravity, blue represents microgravity and red represents hypergravity conditions, (1g, 0g, 2g) denote in-flight gravitational conditions, (1g S, 0g S, 2g S) denote simulated gravitational conditions. *p < 0.05, **p < 0.01, ***p < 0.001.

To evaluate the accuracy of pointing, we looked at the location of hits on the screen with respect to the displayed targets, which we refer to as deviations from the targets. The absolute deviations of hits for each gravitational and simulation condition, averaged for all parabolas and targets, are shown in Figures 2A,D. Additionally, signed deviations of hits for each individual target are presented in Figures 2B,E for microgravity and simulated microgravity and in Figures 2C,F for hypergravity and simulated hypergravity conditions. *Post hoc* tests results are presented in Table 1.

**Table 1:** *Post hoc* analysis for signed deviations of hits.

	Down				Up			
	Comparison	z	p		Comparison	z	p	
0g	1g–0g	2.436	0.104		1g–0g	0.096	1.000	
	1g S–0g S	0.122	1.000		1g S–0g S	−7.757	<0.001	***
	0g S–0g	0.297	1.000		0g S–0g	−5.421	<0.001	***
2g	1g–2g	0.679	1.000		1g–2g	6.976	<0.001	***
	1g S–2g S	−1.058	1.000		1g S–2g S	0.340	1.000	
	2g S–2g	0.709	1.000		2g S–2g	−3.452	0.004	**
1g	1g S–1g	2.470	0.095		1g S–1g	2.733	0.044	*

For downward movements the statistical analysis showed no significant effects of simulation (*F*_(1,278)_ = 3.52, *p* = 0.061), gravity (*F*_(2,278)_ = 2.65, *p* = 0.071) or the interaction of gravity × simulation (*F*_(2,278)_ = 1.40, *p* = 0.245). For the microgravity conditions, no comparisons were significantly different. Despite that, a similar trend is visible where the absolute deviation of hits is slightly higher in microgravity compared to normogravity for both in-flight and simulated conditions. The same effects were observed in hypergravity and simulated hypergravity, where there was a slight increase in the absolute deviations, however, this was not significant. The accuracy of downward movements in the simulated normogravity condition is better, however, this difference is not significant. For all gravitational conditions, we observed a trend in the signed deviations of hits for each target, where the deviations increase with the increased target distance.

For upward movements the statistical analysis showed a significant effect of gravity (*F*_(2,278)_ = 53.34, *p* < 0.001), simulation (*F*_(1,278)_ = 11.21, *p* < 0.001), and the interaction of gravity × simulation (*F*_(2,278)_ = 18.49, *p* < 0.001). Interestingly, for upward movements in microgravity, there was no significant difference in the deviations from normogravity conditions. There was, however, a significant increase in deviations in the simulated microgravity condition in comparison with normogravity or microgravity (0g S–0g, 1g S–0g S). Also, while we observed both positive and negative deviations of hits in normogravity and microgravity, the same was not true for the simulated microgravity where the subjects on average hit above all of the targets (only positive deviations). Concerning hypergravity conditions, the absolute deviations increased significantly compared to normogravity (1g–2g) whereas this was not the case for the simulated hypergravity, where there was only a small increase in the absolute deviations. We also noted marginally higher deviations in the simulated normogravity conditions compared to normogravity (1g S–1g).

### Motion Kinematics

#### Duration of Movements

**Figure 3:**
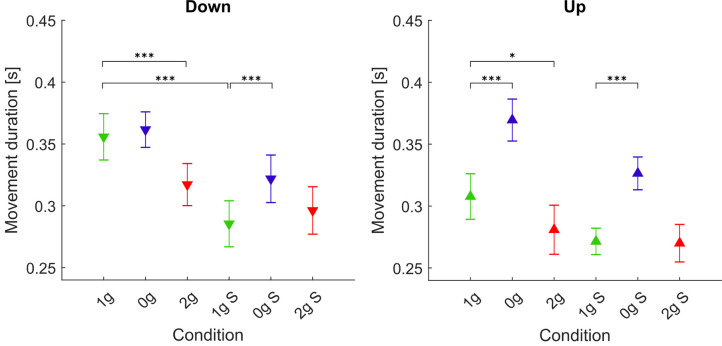
Movement durations in different gravitational and simulation conditions. Downward-pointing triangles denote mean values for downward movements, upward-pointing triangles denote mean values for upward movements, the whiskers denote the standard error of the mean. Green represents normogravity, blue represents microgravity and red represents hypergravity conditions, (1g, 0g, 2g) denote in-flight gravitational conditions, (1g S, 0g S, 2g S) denote simulated gravitational conditions. *p < 0.05, ***p < 0.001.

The durations of movements for each gravitational and simulation condition averaged for all parabolas and targets are shown in Figure 3, whereas *post hoc* tests results are presented in Table 2.

**Table 2:** *Post hoc* analysis for movement duration.

	Down				Up			
	Comparison	z	p		Comparison	z	p	
0g	1g–0g	−0.505	1.000		1g–0g	−8.978	<0.001	***
	1g S–0g S	−4.173	<0.001	***	1g S–0g S	−7.069	<0.001	***
	0g S–0g	2.000	0.318		0g S–0g	2.437	0.104	
2g	1g–2g	5.062	<0.001	***	1g–2g	3.148	0.012	*
	1g S–2g S	−1.238	1.000		1g S–2g S	0.185	1.000	
	2g S–2g	−0.130	1.000		2g S–2g	−1.292	1.000	
1g	1g S–1g	5.881	<0.001	***	1g S–1g	1.455	1.000	

For downward movements the statistical analysis showed a significant effect of gravity (*F*_(2,278)_ = 18.83, *p* < 0.001), simulation (*F*_(1,278)_ = 19.15, *p* < 0.001) and the interaction of gravity × simulation (*F*_(2,278)_ = 9.52, *p* = 0.001). Concerning microgravity conditions, *post hoc* tests revealed that for downward movements there was a significant increase in movement duration compared to normogravity only in the simulated trials (1g S–0g S). The movement durations during real and simulated microgravity (0g–0g S) were not significantly different. Also for hypergravity conditions, there was no significant difference between real and simulated conditions. However, a significant decrease in movement duration was observed during flight (1g–2g) but not during the simulation. Interestingly, movement durations were significantly shorter in the simulated normogravity trials compared to those during flight (1g S–1g).

For upward movements the statistical analysis showed a significant effect of gravity (*F*_(2,278)_ = 109.28, *p* < 0.001) and the interaction of gravity × simulation (*F*_(2,278)_ = 3.71, *p* = 0.026), but not simulation alone (*F*_(1,278)_ = 2.00, *p* = 0.158). *Post hoc* tests revealed that movement duration in microgravity increased significantly both during flight (1g–0g) and in simulation (1g S–0g S). Movement duration during real microgravity did not significantly differ from the simulated condition (0g S–0g). Movement duration during hypergravity was significantly lower compared to normogravity only in the real condition (1g–2g). No statistical difference was found when comparing both normogravity conditions (1g–1g S).

#### Velocity Profiles and Maximum Velocity

**Figure 4:**
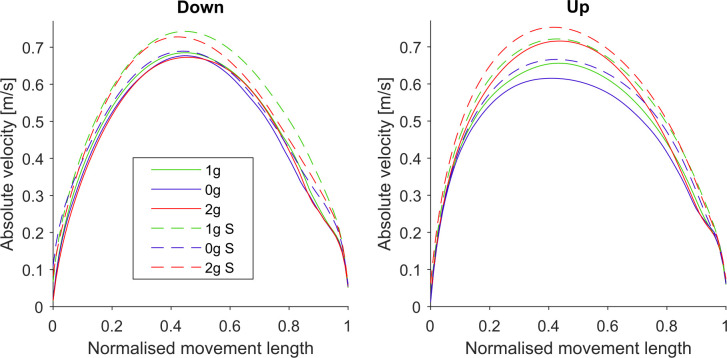
Mean velocity profiles for downward and upward movements in all conditions. Solid lines represent the conditions during flight (1g, 0g, 2g) whereas dashed lines represent the simulated conditions (1g S, 0g S, 2g S). Green represents normogravity, blue represents microgravity and red represents hypergravity conditions.

**Figure 5:**
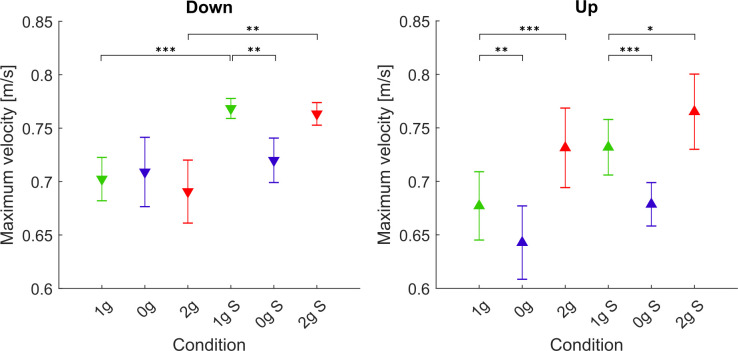
Maximum velocities. Downward-pointing triangles denote mean values for downward movements, upward-pointing triangles denote mean values for upward movements, the whiskers denote the standard error of the mean. Green represents normogravity, blue represents microgravity and red represents hypergravity conditions, (1g, 0g, 2g) denote in-flight gravitational conditions, (1g S, 0g S, 2g S) denote simulated gravitational conditions. *p < 0.05, **p < 0.01, ***p < 0.001.

Figure 4 shows the mean velocity profiles for all gravitational and simulation conditions normalized for target distance in the y direction. Here we can see that the shape of velocity profiles remained constant throughout the various gravitational conditions, i.e., the peaks did not shift, which we had already evaluated when analyzing the TPV parameter. However, we observed a change in the magnitude of the velocity profiles especially for the upward movements where the maximum velocities increased in hypergravity conditions. In order to more clearly illustrate these changes, we present the maximum velocities for each gravitational and simulation condition, averaged for all parabolas and targets in Figure 5. *Post hoc* tests results are presented in Table 3.

**Table 3:** *Post hoc* analysis for maximum velocity.

	Down				Up			
	Comparison	z	p		Comparison	z	p	
0g	1g–0g	−1.005	1.000		1g–0g	3.260	0.008	**
	1g S–0g S	3.713	<0.001	**	1g S–0g S	4.323	<0.001	***
	0g S–0g	0.455	1.000		0g S–0g	−0.079	1.000	
2g	1g–2g	−0.601	1.000		1g–2g	−5.206	<0.001	***
	1g S–2g S	0.387	1.000		1g S–2g S	−2.693	0.050	*
	2g S–2g	−3.346	0.006	**	2g S–2g	0.553	1.000	
1g	1g S–1g	4.334	<0.001	***	1g S–1g	−1.540	0.865	

Statistical analysis for downward movements showed a significant main effect of simulation (*F*_(1,278)_ = 15.69, *p* < 0.001) as well as the interaction of gravity × simulation (*F*_(2,278)_ = 6.68, *p* = 0.002), whereas there was no significant main effect of gravity (*F*_(2,278)_ = 2.12, *p* = 0.122). In microgravity conditions, the maximum velocity was significantly lower only in the simulated condition. However, there was no significant difference between the actual and simulated microgravity conditions (0g S–0g). In contrast, the maximum velocity in simulated hypergravity was higher compared with the real hypergravity condition (2g S–2g). Similarly, the maximum velocity in normogravity conditions was higher in the simulated trials compared to those during flight (1g S–1g).

For upward movements, analyzing the maximum velocity revealed a significant main effect of gravity (*F*_(2,278)_ = 54.85, *p* < 0.001). No significant effects of simulation (*F*_(1,278)_ = 0.54, *p* = 0.462) or interaction of gravity × simulation (*F*_(2,278)_ = 1.24, *p* = 0.292) were found. Concerning microgravity conditions, the maximum velocity was significantly lower than in normogravity both in flight and on the ground (1g–0g, 1g S–0g S). There was no significant difference between simulated and real microgravity conditions (0g S–0g). For hypergravity conditions, the maximum velocity increased in both real and simulated conditions (1g–2g, 1g S–2g S) and there was no difference between the two hypergravity conditions (2g S–2g). The maximum velocity in simulated normogravity was higher than in-flight, however, this difference was not statistically significant (1g S–1g).

#### Shape of Trajectories

**Figure 6:**
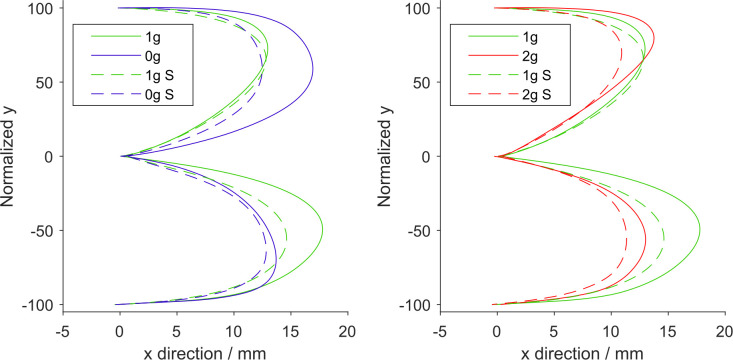
Mean trajectories normalized for target distance for different gravitational and simulation conditions. Left: normogravity and microgravity conditions, Right: normogravity and hypergravity conditions. All trajectories start at the coordinate (0, 0) and end at either (0, 100) for upward movements or (0, −100) for downward movements. Solid lines represent the conditions during flight (1g, 0g, 2g) whereas dashed lines represent the simulated conditions (1g S, 0g S, 2g S). Green represents normogravity, blue represents microgravity and red represents hypergravity conditions.

Figure 6 shows the mean trajectories for all gravitational and simulation conditions normalized for target distance in the y direction. Here we can observe an increased displacement in the x direction in microgravity for upward movements, whereas for the downward movements we can see a decreased displacement in the x direction both in microgravity and hypergravity.

**Figure 7:**
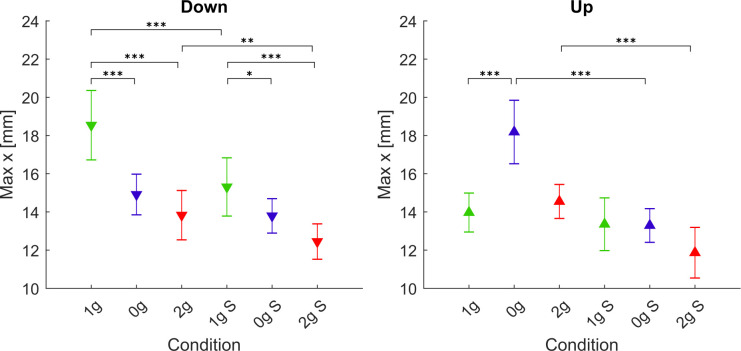
Maximum displacement in the x direction. Downward-pointing triangles denote mean values for downward movements, upward-pointing triangles denote mean values for upward movements, the whiskers denote the standard error of the mean. Green represents normogravity, blue represents microgravity and red represents hypergravity conditions, (1g, 0g, 2g) denote in-flight gravitational conditions, (1g S, 0g S, 2g S) denote simulated gravitational conditions. *p < 0.05, **p < 0.01, ***p < 0.001.

In order to more clearly compare the differences between conditions, we present the maximum normalized displacements in x direction for each gravitational and simulation condition, averaged for all parabolas and targets, in Figure 7. The *post hoc* tests results for the maximum displacements in x direction are presented in Table 4.

**Table 4:** *Post hoc* analysis for maximum displacement in the x direction.

	Down				Up			
	Comparison	z	p		Comparison	z	p	
0g	1g–0g	7.687	<0.001	***	1g–0g	−8.430	<0.001	***
	1g S–0g S	2.892	0.027	*	1g S–0g S	0.117	1.000	
	0g S–0g	2.689	0.050		0g S–0g	9.707	<0.001	***
2g	1g–2g	9.427	<0.001	***	1g–2g	−1.270	1.000	
	1g S–2g S	5.459	<0.001	***	1g S–2g S	2.649	0.057	
	2g S–2g	3.715	0.001	**	2g S–2g	5.664	<0.001	***
1g	1g S–1g	6.882	<0.001	***	1g S–1g	1.814	0.488	

For downward movements, the statistical analysis showed a significant effect of gravity (*F*_(2,278)_ = 64.02, *p* < 0.001), simulation (*F*_(1,278)_ = 55.62, *p* < 0.001) and the interaction of gravity × simulation (*F*_(2,278)_ = 4.95, *p* = 0.008). In microgravity conditions, *post hoc* tests revealed that for downward movements there was a significant decrease in maximum x displacement for both the real and simulated trials (1g–0g, 1g S–0g S). The comparison of real and simulated microgravity conditions was not significantly different (0g S–0g), albeit with a p-value of 0.05. For hypergravity conditions, the maximum x displacement was lower than in normogravity for both real and simulated conditions (1g–2g, 1g S– 2g S). At the same time, the displacement was significantly lower in the simulated hypergravity compared with the in-flight condition (2g S–2g). Similarly, as with what we observed with the movement duration, the maximum x displacement in normogravity was lower in the simulated trials compared to those in-flight (1g S–1g).

For upward movements the statistical analysis showed a significant effect of gravity (*F*_(2,278)_ = 29.90, *p* < 0.001), simulation (*F*_(1,278)_ = 90.97, *p* < 0.001) and the interaction of gravity × simulation (*F*_(2,278)_ = 16.16, *p* < 0.001). *Post hoc* tests for microgravity conditions revealed that the maximum x displacement increased significantly only during flight (1g–0g). The maximum x displacement in the simulated microgravity appears to be unchanged from normogravity. This is also reflected in a significant difference between the real and simulated 0g condition (0g S–0g). Maximum x displacement during hypergravity or simulated hypergravity was not significantly different compared to normogravity. However, a significant difference between real and simulated hypergravity (2g S–2g) conditions was observed. No statistical difference was found when comparing both normogravity conditions for the upward movements.

#### Movement Asymmetry: Relative Time to Peak Velocity (TPV)

**Figure 8:**
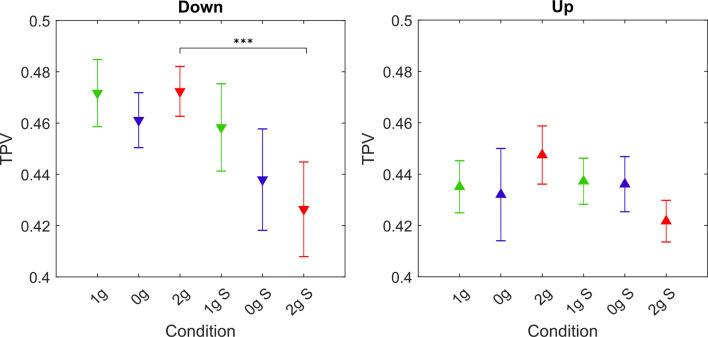
Values of the TPV parameter for different gravitational and simulation conditions. Downward-pointing triangles denote mean values for downward movements, upward-pointing triangles denote mean values for upward movements, the whiskers denote the standard error of the mean. Green represents normogravity, blue represents microgravity and red represents hypergravity conditions, (1g, 0g, 2g) denote in-flight gravitational conditions, (1g S, 0g S, 2g S) denote simulated gravitational conditions. ***p < 0.001.

As a measure of the asymmetry of movement, we calculated the normalized TPV. The TPV for each gravitational and simulation condition averaged for all parabolas and targets, are shown in Figure 8, whereas *post hoc* tests results are presented in Table 5.

**Table 5:** *Post hoc* analysis for TPV.

	Down				Up		
	Comparison	z	p		Comparison	z	p	
0g	1g–0g	1.067	1.000		1g–0g	0.395	1.000
	1g S–0g S	1.708	0.613		1g S–0g S	0.115	1.000
	0g S–0g	2.182	0.204		0g S–0g	−1.214	1.000
2g	1g–2g	−0.160	1.000		1g–2g	−1.249	1.000
	1g S–2g S	2.678	0.052		1g S–2g S	1.549	0.850
	2g S–2g	4.332	<0.001	***	2g S–2g	1.786	0.519
1g	1g S–1g	1.430	1.000		1g S–1g	−0.972	1.000

For downward movements the statistical analysis showed a significant effect of simulation (*F*_(1,278)_ = 20.16, *p* < 0.001), whereas the effect of gravity and the interaction of gravity × simulation were not significant (*F*_(2,278)_ = 2.30, *p* = 0.102) and (*F*_(2,278)_ = 2.31, *p* = 0.101) respectively. Concerning microgravity conditions, *post hoc* tests revealed no significant differences between conditions. For hypergravity conditions, TPV significantly decreased in the simulated condition (2g S–2g). No changes were observed between normogravity conditions.

For upward movements the statistical analysis showed no significant effects of gravity (*F*_(2,278)_ = 0.09, *p* = 0.912) and simulation (*F*_(1,278)_ = 0.04, *p* = 0.839), but it did reveal a significant interaction effect of gravity × simulation (*F*_(2,278)_ = 3.12, *p* = 0.046) although the p-value was barely significant. The results of *post hoc* tests for the upward movements reflect the absence of significant effects.

### Muscle Activity

**Figure 9:**
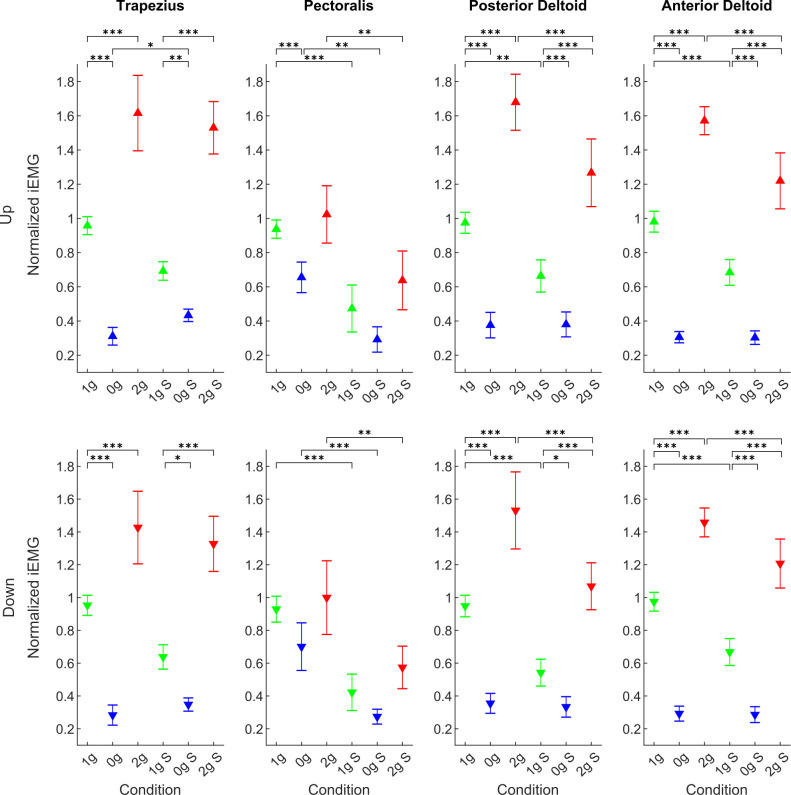
Normalized integrated EMG for the Trapezius, Pectoralis, Anterior Deltoid, and Posterior Deltoid for all conditions. Downward-pointing triangles denote mean values for downward movements, upward-pointing triangles denote mean values for upward movements, the whiskers denote the standard error of the mean. Green represents normogravity, blue represents microgravity and red represents hypergravity conditions, (1g, 0g, 2g) denote in-flight gravitational conditions, (1g S, 0g S, 2g S) denote simulated gravitational conditions. *p < 0.05, **p < 0.01, ***p < 0.001.

The normalized iEMG of all muscles, for each gravitational and simulation condition, averaged for all parabolas and targets, are presented in Figure 9. The mean values for downward movements are denoted with downward- pointing triangles and mean values of upward movements are denoted with upward-pointing triangles.

The ANOVA revealed a significant main effect of gravity for downward movements for the Trapezius (*F*_(2,246)_ = 152.99, *p* < 0.001), Pectoralis (*F*_(2,246)_ = 12.60, *p* < 0.001), Anterior Deltoid (*F*_(2,246)_ = 535.16, *p* < 0.001), and Posterior Deltoid (*F*_(2,246)_ = 185.83, *p* < 0.001). A significant effect of simulation was found only for the Pectoralis (*F*_(1,246)_ = 43.33, *p* < 0.001), Anterior Deltoid (*F*_(1,246)_ = 24.66, *p* < 0.001) and Posterior Deltoid (*F*_(1,246)_ = 16.78, *p* < 0.001) while there was no effect at the Trapezius muscle (*F*_(1,246)_ = 0.54, *p* = 0.461). Most importantly, there was a significant interaction effect of gravity × simulation for the Trapezius (*F*_(2,246)_ = 4.55, *p* = 0.011), Anterior Deltoid (*F*_(2,246)_ = 11.13, *p* < 0.001) and Posterior Deltoid (*F*_(2,246)_ = 9.93, *p* < 0.001), while there was no significant effect at the Pectoralis muscle (*F*_(2,246)_ = 0.31, *p* = 0.730). *Post hoc* test results of the different conditions are presented in Table 6.

**Table 6:** *Post hoc* analysis for iEMG.

		Down				Up			
	Comparison	z	p		Comparison	z	p		
Trapezius	0g	1g–0g	8.086	<0.001	***	1g–0g	9.387	<0.001	***
		1g S–0g S	3.188	0.010	*	1g S–0g S	3.365	0.005	**
		0g S–0g	−1.494−	0.946		0g S–0g	−2.711−	0.047	*
	2g	1g–2g	−5.789−	<0.001	***	1g–2g	−9.345−	<0.001	***
		1g S–2g S	−7.572−	<0.001	***	1g S–2g S	−10.868−1	<0.001	***
		2g S–2g	0.135	1.000		2g S–2g	−0.147−	1.000	
	1g	1g S–1g	2.679	0.052		1g S–1g	2.465	0.096	
Pectoralis	0g	1g–0g	2.665	0.054		1g–0g	3.877	<0.001	***
		1g S–0g S	1.719	0.600		1g S–0g S	2.192	0.199	
		0g S–0g	3.841	<0.001	***	0g S–0g	3.440	0.004	**
	2g	1g–2g	−0.780−	1.000		1g–2g	−1.051−	1.000	
		1g S–2g S	−1.760−	0.549		1g S–2g S	−1.988−	0.328	
		2g S–2g	3.417	0.004	**	2g S–2g	3.651	0.002	**
	1g	1g S–1g	4.533	<0.001	***	1g S–1g	4.769	<0.001	***
Posterior Deltoid	0g	1g–0g	8.512	<0.001	***	1g–0g	10.570	<0.001	***
		1g S–0g S	2.765	0.040	*	1g S–0g S	4.463	<0.001	***
		0g S–0g	−1.109−	1.000		0g S–0g	−1.386−	1.000	
	2g	1g–2g	−8.595−	<0.001	***	1g–2g	−12.139−1	<0.001	***
		1g S–2g S	−6.967−	<0.001	***	1g S–2g S	−9.512−	<0.001	***
		2g S–2g	4.561	<0.001	***	2g S–2g	5.105	<0.001	***
	1g	1g S–1g	3.893	<0.001	
***	1g S–1g	3.745	0.001	
**
Anterior Deltoid	0g	1g–0g	15.6321	<0.001	***	1g–0g	17.424	<0.001	***
		1g S–0g S	7.917	<0.001	***	1g S–0g S	8.717	<0.001	***
		0g S–0g	−0.776−	1.000		0g S–0g	−1.379−	1.000
	2g	1g–2g	−11.2781−	<0.001	***	1g–2g	−14.970−1	<0.001	***
		1g S–2g S	−11.1901−	<0.001	***	1g S–2g S	−12.246−1	<0.001	***
		2g S–2g	4.213	<0.001	***	2g S–2g	6.808	<0.001	***
	1g	1g S–1g	5.464	<0.001	***	1g S–1g	5.671	<0.001	***

For the upward direction, the effects were exactly the same. We found a significant main effect of gravity for the Trapezius (*F*_(2,244)_ = 274.77, *p* < 0.001), Pectoralis (*F*_(2,244)_ = 21.48, *p* < 0.001), Anterior Deltoid (*F*_(2,244)_ = 712.69, *p* < 0.001) and Posterior Deltoid (*F*_(2,244)_ = 340.82, *p* < 0.001). A significant effect of simulations was found for the Pectoralis (*F*_(1,244)_ = 44.55, *p* < 0.001), Anterior (*F*_(1,244)_ = 38.41, *p* < 0.001) and Posterior Deltoid (*F*_(1,244)_ = 17.35, *p* < 0.001), but not the Trapezius (*F*_(1,244)_ = 0.03, *p* = 0.851). Analogous to the downward direction there was a significant interaction of gravity × simulation for the Trapezius (*F*_(2,244)_ = 6.83, *p* = 0.001), Anterior Deltoid (*F*_(2,244)_ = 20.02, *p* < 0.001) and Posterior Deltoid (*F*_(2,244)_ = 11.87, *p* < 0.001), while there was no significant effect at the Pectoralis muscle (*F*_(2,244)_ = 0.49, *p* = 0.609). *Post hoc* test results of the different conditions are presented in Table 6.

For both directions and for all muscles we observed a decrease in muscle activity in both the real and simulated microgravity conditions compared to normogravity, whereas in the real and simulated hypergravity conditions we observed an increase in muscle activity compared to normogravity conditions.

## Discussion

During the parabolic flights, the subjects experienced changes in gravitational conditions that affected their whole body including the vestibular system. The forces exerted on the limbs of the individuals were continuous and acted on the entirety of the limb. Additionally, the stylus that the subjects were manipulating in order to perform the task was also subjected to these gravitational changes. On the other hand, in the trials on the ground, the subjects experienced only a locally applied force on the wrist that simulated the same torque in the shoulder joint the subjects experienced during microgravity and hypergravity during flight. Comparing these two conditions, we aimed to answer our main question. Can we elicit the same changes in movement parameters only with simulating local gravity conditions on the arm compared to full body micro- and hypergravity conditions?

### Effects on the Task Outcome

Looking at the task outcome, we noted a trend of increased hit deviations (reduced accuracy) in microgravity and hypergravity for upward and downward movements both in-flight and in simulation. This is in line with other studies which observed a decreased accuracy in these conditions (Bock et al., 1992; Fisk et al., 1993; Bringoux et al., 2012). However, these changes in deviations were only significant for the upward movements. We could not mimic the changes in hypergravity with our simulation, the subjects seemed to be better able to compensate for the force exerted on the wrist than when dealing with full-body hypergravity conditions. At first glance, we saw an increase in the absolute hit deviations in the simulated microgravity which coincided with the increased deviations observed in microgravity. However, the inspection of the signed deviations per target (Figure 2B) revealed that the simulated microgravity condition affected the accuracy of subjects in a different way. Namely, in the 0g S condition, subjects overshot all of the targets whereas in microgravity, as well as in both normogravity conditions, the targets further away from the starting position (4–7) were mostly undershot. This shows, that the subjects were better able to adapt to microgravity conditions during a flight than in our simulation setup. Since the setup could not provide an adequate representation of microgravity conditions, namely the vestibular system and manipulated object were unaffected, it is probable that this resulted in some sort of sensory conflict that prevented the subjects to complete the task in the same way as in normogravity.

### Effects on Movement Kinematics

Our analysis of the movement kinematics parameters showed that (compared to normogravity) the duration of movements increased in microgravity (0g and 0g S) for both upward and downward movements. The only exception being the downward movements in-flight (0g) where the increase in movement duration was not significant. On the other hand, the movement durations were lower in hypergravity in-flight for both directions of movement, but there was no change in movement duration in the simulation trials. These results are mostly consistent with other studies, where they also noted an increase of movement duration in microgravity and a decrease in hypergravity (Bock et al., 1996; Papaxanthis et al., 2005; Macaluso et al., 2016). Interestingly, movement durations for downward movements were shorter in the simulated normogravity trials compared to those during flight (1g S vs. 1g). Regarding the shape of the trajectories, we found an increased displacement in the x direction only in microgravity for the upward movements in-flight while there was no change during simulation. This could be explained by the fact that during flight the whole environment was isotropous which meant that subjects had to more actively refrain from moving sideways. In contrast, for the downward movements, we observed a decrease in displacement in the x direction in both microgravity and hypergravity in-flight and in the simulated trials. Compared to the upward movements, the task stability may have improved when performing downward movements which decreased the displacement in the x direction.

### Changes in Velocity Profiles

The shape of the velocity profiles did not vary across different gravitational or simulation conditions which we showed with the analysis of the TPV parameter. However, we did note some changes in the maximum velocities. Namely, the maximum velocity was lower in microgravity and higher in hypergravity for the upward movements both in-flight and in simulation. For the downward movements, however, there were no differences in flight, but in simulation, the maximum velocity was lower for the 0g S condition. Additionally, we observed that the TPV parameter remained largely unaffected by the different gravitational conditions both in-flight and in simulation. However, we noted a lower TPV in the simulated hypergravity compared to the in-flight hypergravity (2g S vs. 2g).

### Changes in Muscle Activity

The analysis of muscle activity showed, that the Pectoralis muscle was the least affected by gravitational conditions both in flight and during simulation. This is probably because it acts primarily perpendicular to the gravitational vector. However, we still noted a significant decrease of activity in microgravity in-flight (0g). For all other muscles we observed the same results, where for both directions, the muscle activity was significantly increased in real and simulated hypergravity conditions and lowered in the microgravity conditions. This is comparable with other studies with pointing tasks performed on parabolic flights (Chen et al., 1999), where EMG activity increased in hypergravity and decreased in microgravity. Our results, therefore, show, that the simulations of gravitational environments with our system were quite good from an action point of view.

### Could On-Ground Training With Simulated Gravitational Conditions be Beneficial?

Apart from the trajectory shape, the locally simulated microgravity and hypergravity conditions of the limb appear to have a similar effect on the kinematic parameters analyzed. That is, increased movement durations in microgravity, decreased maximum velocities in microgravity, and increased in hypergravity as well as an unaffected asymmetry of movement. Additionally, the muscle activity was lower in microgravity and higher in hypergravity conditions. This indicates that training in normogravity conditions with only locally simulated microgravity or hypergravity could be beneficial for training arm reaching movements in microgravity and hypergravity (Papaxanthis et al., 1998). The discrepancy between the changes in the maximal x displacement and movement durations could potentially stem from the fact that our active support system had contact only at the wrist. Such a simulation of microgravity did not have the full effect as during the parabolic flight, it did however elicit similar responses when looking at the duration of movements. This also demonstrates that proprioceptive feedback provides a lot of relevant information to control kinematics. Perhaps analyzing the impact of an exoskeleton device with a distributed whole limb gravity compensation would be interesting. A distributed unloading of the arm could potentially provide better feedback to the subjects and hence provide a better environment for training movements in microgravity and hypergravity.

In Bringoux et al. (2012) they observed reduced accuracy of subjects in both microgravity and hypergravity conditions. In microgravity, their accuracy was restored to normal when they applied a gravity-like torque before and during the movements performed. Their results suggest that arm motor planning and control are tuned with respect to gravitational information issued from joint torque. Similar conclusions were found by Rousseau et al. (2016), where they showed that information coming from the initial state of the sensorimotor system is determinant to planning movements in the gravity field. However, in these studies, the subjects were not manipulating any object with their hand as was the case in our experiment. During the microgravity condition, the subjects did not feel any weight at the stylus while maintaining the position at the starting position. The motor system is likely to interpret this as the absence of mass and resultantly reduces the motor command for the movements. However, the motor command to accelerate the stylus should remain unchanged because the mass is not changed, slowing the movement and increasing the movement duration. In our simulated microgravity conditions, the object manipulated was still affected by gravity thereby creating a sensory conflict in the estimation of the arm and object dynamics. While we still observed similar changes in 0g and 0g S (e.g., increases movement duration), the analysis of the deviations of target hits revealed that the subjects consistently overshot the targets, which resulted in a worse performance in the simulated microgravity (0g S) compared to the microgravity (0g) condition. Notably, our observed changes in movement duration and maximum velocity in the simulated microgravity conditions are in contrast with some other studies that analyzed changes in kinematic features of arm movements in normogravity with weight support systems (Prange et al., 2009; Coscia et al., 2014), where such changes were not present. However, in these studies, movements were not limited to vertical arm movements which might be the cause of the differences in the observed results. Similarly, in a parabolic flight study where subjects performed pointing movements predominantly in the horizontal direction no significant differences between gravitational conditions were observed for movement duration and accuracy of pointing (Artiles et al., 2018).

Overall, we showed that locally simulated gravity alterations can elicit similar changes in movement characteristics for arm reaching movements and could potentially be used as a means of training individuals prior to undertaking tasks in changed gravitational conditions.

## Data Availability Statement

The raw data supporting the conclusions of this article will be made available by the authors, without undue reservation.

## Ethics Statement

The studies involving human participants were reviewed and approved by French National Ethics committee (2018-A03379-46). The patients/participants provided their written informed consent to participate in this study.

## Author Contributions

GB, DN, CP, OW, and JB designed the study. MJ, JB, OW, and GB performed the experiment. MJ and TK analyzed the data and wrote the manuscript. OW, JB, GB, DN, CP, and TK gave feedback on the manuscript. MJ, TK, GB, DN, CP, OW, and JB read and approved the submitted version. All authors contributed to the article and approved the submitted version.

## Conflict of Interest

The authors declare that the research was conducted in the absence of any commercial or financial relationships that could be construed as a potential conflict of interest.

## Publisher’s Note

All claims expressed in this article are solely those of the authors and do not necessarily represent those of their affiliated organizations, or those of the publisher, the editors and the reviewers. Any product that may be evaluated in this article, or claim that may be made by its manufacturer, is not guaranteed or endorsed by the publisher.
